# Anti-inflammatory Properties of Strontium Oxide Nanoparticles Synthesized From Suaeda monoica Saltmarsh

**DOI:** 10.7759/cureus.56355

**Published:** 2024-03-18

**Authors:** Pranam Sinha, Priya Boopathi, Vasugi Suresh, Sivaperumal Pitchiah

**Affiliations:** 1 Physiology, Saveetha Dental College and Hospital, Saveetha Institute of Medical and Technical Sciences, Saveetha University, Chennai, IND; 2 Prosthodontics, Saveetha Dental College and Hospital, Saveetha Institute of Medical and Technical Sciences, Saveetha University, Chennai, IND

**Keywords:** strontium oxide nanoparticles, biosynthesis, anti-inflammatory, uv-spectroscopy, suaeda monoica

## Abstract

Background

Currently, nanotechnology is a rapidly advancing field of research. Because of their nanoscale dimensions, nanoparticles (NPs) find application in a wide range of industries, including engineering and medicine. The leaves of *Suaeda monoica* have anti-inflammatory qualities. The purpose of this study was to create SrO NPs isolated from the leaves of *S. monoica* aqueous extract and to evaluate their anti-inflammatory efficacy. The *S*. *monoica* saltmarsh, commonly known as South-Indian Seepweed, is a mangrove-associated plant and has been used as traditional medicine for decades with multifunctional biological activity.

Objectives

The aim of our study is to biosynthesize strontium oxide NPs from *S. monoica* saltmarsh and to see whether they have any anti-inflammatory properties.

Materials and methods

In the present study, the pharmacological significance was studied using crude extract and synthesized SrO NPs from *S. monoica. *The synthesized SrO NPs were characterized using UV spectrophotometry. The *in vitro *anti-inflammatory assay was analyzed using egg albumin denaturation. SrO NPs' peak observance was found at 630 nm, and a graph was plotted for the zone of inhibition vs concentration and compared with the standard.

Results

It was observed that the color of the SrO NPs deepened during the synthesis process. Furthermore, at a wavelength of 630 nm, the UV spectrum analysis showed a noteworthy absorption value of 1.4. The activity of inflammatory enzymes is significantly impacted by the anti-inflammatory properties of SrO NPs in the protein denaturation inhibition test.

Conclusions

The application of SrO NPs in the synthesis process has the potential to enhance the anti-inflammatory activity of *Suaeda monoica *as evidenced by the observed increase in anti-inflammatory capacity and defense against infections and injury.

## Introduction

Nanoparticles (NPs) have drawn a lot of attention in recent years for their predicted impact on numerous fields, including energy, medicine, and electronics. There is presently an increasing demand to offer an environmentally friendly method of creating NPs, as using toxic substances in the synthesis pathway causes significant problems for the environment [1]. The prospect of implementing therapeutic NPs in the detection and treatment of human tumors has been highlighted by the rapidly evolving field of nanoscience. Because they differ from conventional small-molecule medications in terms of their size and structure, nanoscale particles and molecules represent a viable alternative for the treatment of medical conditions. The US Food and Drug Administration (FDA) has approved numerous drug makers in recent years for the development of medications based on NPs [2]. The inflammatory response is a major pathogenic factor in several illnesses. Anti-inflammatory drug therapy is beneficial for treating diseases therapeutically. Anti-inflammatory cytokines, on the other hand, include IL-4, IL-10, IL-13, IFN-α, and the transforming growth factor [3-7].

Different techniques can be used to synthesize NPs. The most commonly used processes for creating NPs are chemical ones. Since NPs of noble metals like gold, silver, and platinum are frequently used in regions where people come into contact with them, there is a growing requirement to create ecologically acceptable NP synthesis procedures that don't include dangerous chemicals. As alternatives to chemical and physical approaches, biological methods of NP manufacturing involving microorganisms, enzymes, and plant extracts have been proposed. As opposed to other biological processes, using plants for the creation of NPs can be advantageous because it doesn't involve the time-consuming task of sustaining cells [8]. As a result, research into plant systems as possible biofactories has increased interest in the creation of biological NPs [2-9]. Strontium is naturally found in the minerals strontianite and celestite, which can be obtained through mining. There are a variety of strontium-based nanomaterials, such as doped strontium-based materials, strontium-based oxides, strontium sulfides, and strontium-based nanocomposites [10].

People are turning more and more to natural and holistic methods of health and wellbeing, especially those found in traditional medicine, which means that the usage of plant-based therapies is growing in popularity [11]. The plant has necessary therapeutic qualities, such as hepatoprotection and anti-inflammatory actions, that can have positive health consequences [12]. Natural goods have become essential resources in the last two decades for a range of applications, including the biopesticide and cosmetics industries, as well as the prevention and treatment of microbial disorders [13]. *Suaeda*, a genus of plants in the *Chenopodiaceae* family of saltmarsh plants that comprises small trees and annual and perennial herbs, is also known as a seepweed. The *Suaeda* genus contains roughly 110 unique species. They can be found all over the world on saline and alkaline land, as well as in deserts, lakes, and lakeshores. *Suaeda monoica* possesses anti-inflammatory, hypolipidemic, cardiotonic, antioxidant, antibacterial, and anti-cancer properties [14]. *Suaeda maritima (L)* *Dumort, Suaeda monoica Forssk. ex J.F.Gmel., Suaeda pruinosa Lange, Suaeda maritima subsp. salsa (L.) Soó and Suaeda vermiculata* *Forssk *are accessible in Indian coastal regions but have gotten very little research [15]. The plant kingdom is made up of a diverse group of plants that have useful therapeutic compounds that are biologically active [3,4].

Numerous plant-derived compounds have been effectively extracted, identified, and brought into global markets by drug companies. The mangrove herb *Suaeda monoica *(*Chenopodiaceae*) has been used for centuries to treat sore throats, rheumatism, asthma, snake bites, skin illnesses, ulcers, hepatotoxicity, and microbiological infections. Furthermore, its fatty acids, resins, tannins, coumarins, cardiac glycosides, flavonoids, saponins, alkaloids, polyphenols, resins, saponins, and saponins have been identified as medicinal phytoconstituents. It is utilized for the treatment of hepatitis in traditional therapies. *S. monoica* is also applied as an ointment to aid in the healing of wounds. Experimental investigation has revealed that *S. monoica* has antiviral and hepatoprotective properties and antioxidant processes [3].

More specific cells (B and T cells) work with the adaptive immune system to produce particular antibodies and receptors that kill cancer cells and other invasive invaders [16]. Everything was predicated on experiences because there was not sufficient knowledge available at the time concerning the root causes of the ailments or the particular plants that could be used as a remedy [17]. Positive surface potential combined with their size and morphology can create functional NPs that have strong antibacterial and anti-inflammatory properties [18]. This study characterizes the green synthesis of strontium NPs using *S. monoica* powder extract through UV-visible spectroscopy and also investigates their bioactivities, such as anti-inflammatory assays.

## Materials and methods

Preparation of *S. monoica* powder

**Figure 1:**
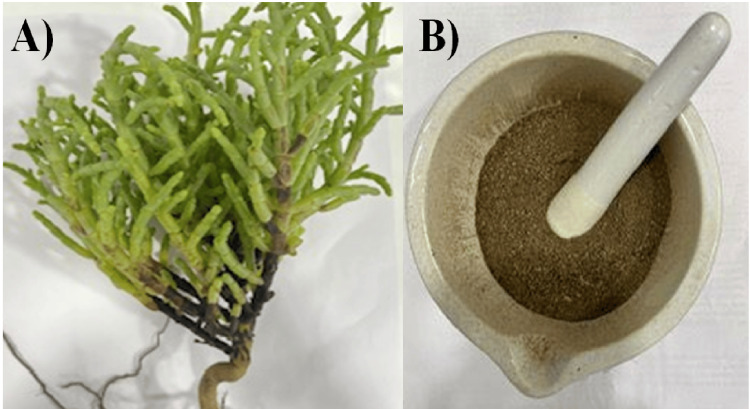
S. monoica saltmarsh plant sample (A) and S. monoica plant powder sample (B)

*S. monoica* was collected based on medicine from the marine areas of Tamil Nadu. The collected plant samples were harvested and washed under sterile distilled water. The samples were shade-dried at room temperature. The dried plant materials were then ground to a fine powder (Figure 1).

Preparation of the aqueous extract of *S. monoica* leaf powder

**Figure 2:**
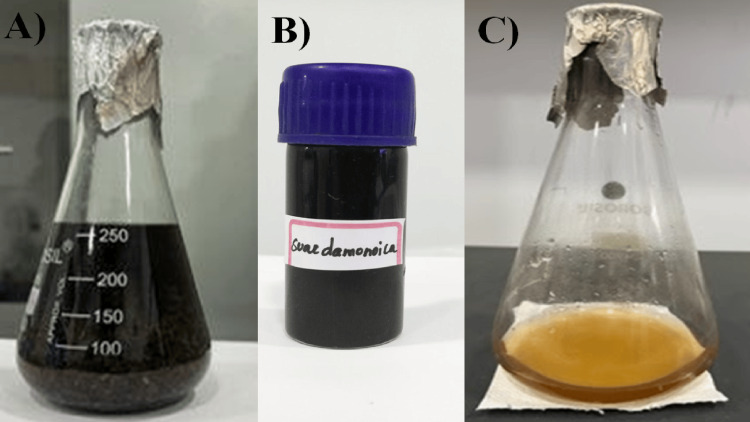
Aqueous extract of S. monoica leaf (A), crude extract (B), and initial stage of nanoparticle synthesis (C)

The dry powder of the plant material, weighing around 35 g, was dissolved in 600 ml of distilled water. Then it was boiled for 30 minutes at 70°C while stirring constantly. Whatman No. 1 filter paper was used to filter the supernatant, and the resulting filtrate was utilized to make SrO NPs, which were used subsequently (Figure 2).

Synthesis of strontium oxide nanoparticles (SrO_2_ NPs)

An aqueous solution of 1 mmol SrCl_2._6H_2_O was prepared as a stock solution to prepare strontium oxide NPs. 50 ml of *S. monoica* extract (the extract's pH was adjusted to 10 by adding 0.1 mmol NaOH) was combined with 50 ml of strontium stock solution, and the mixture was continuously stirred for 30 minutes at room temperature. A progressive change in hue from light gray to silvery white indicated the development of SrO NPs. The mixture was centrifuged for 15 minutes at 8000 rpm to extract the SrO. After three rounds of washing in deionized water, the NPs were dried in a desiccator filled with calcium carbonate [19].

Characterization of SrO_2 _NPs

Ultraviolet-Visible Spectroscopy

An ultraviolet-visible spectrometer, which is frequently used for nanoparticle characterization and the identification of the surface plasmon resonance property (SPR) of SrO NPs, among other uses, was employed to analyze the SrO NPs. The spectra between 300 and 700 nm were scanned to determine the highest absorption [20].

Anti-inflammatory Activity

In order to generate the reaction mixture (0.5 mL), 0.45 mL of bovine serum albumin (1 percent aqueous solution) was added to different quantities of aspirin, SrO NPs, and aqueous extract (5, 10, 20, 25, 50, and 100 μg/mL). The pH of the reaction mixture was reduced to 6.3 with a small quantity of HCl. The samples were then heated for 20 minutes at 60°C to finish the process, after which they were incubated for 20 minutes at 37°C. Using spectrophotometric equipment, the turbidity of the samples was measured spectrophotometrically at 660 nm after cooling. To find the percentage of protein denaturation that was avoided, apply the formula below: Inhibition% is equal to A control-A sample/A control times 100, where the absorbance of a solution without a test sample serves as a control [21].

Protein Denaturation Inhibition

0.06 mg of trypsin, 1 mL of Tris HCl buffer (20 mM) (pH 7.4), and 1 mL of the test sample at various concentrations were all included in the reaction mixture (2 mL). One milliliter (0.8% by weight to volume) of casein was added to the mixture after it had been incubated for five minutes at 37°C. The combination was allowed to incubate at room temperature for another 20 minutes. To finish the process, 2 milliliters of 70% perchloric acid were added. After centrifuging the hazy suspension, the absorbance of the supernatant at 210 nm was calculated and compared to that of a blank buffer. In this instance, the previously stated formula was used to determine the percentage inhibition of proteinase activity [21].

## Results

UV visible spectroscopy of SrO_2 _NPs

**Figure 3:**
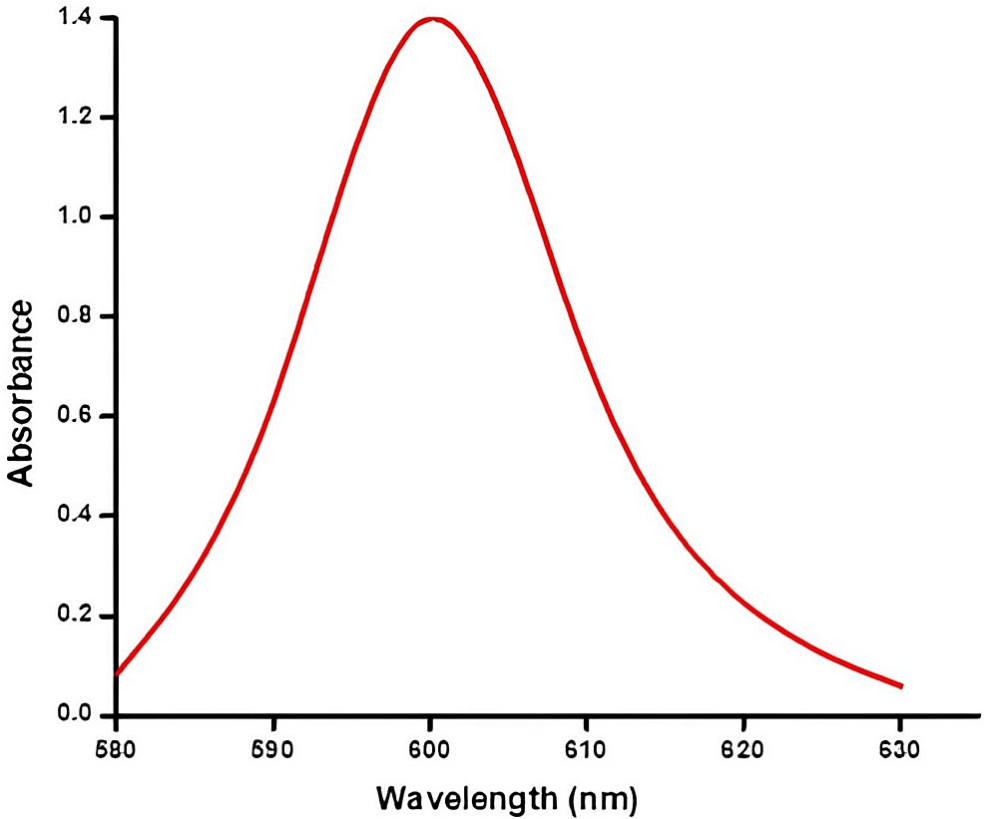
UV visible spectroscopy of strontium oxide nanoparticles from S. monoica UV: Ultraviolet, *S. monoica: Suaeda monoica*

Figure 3 demonstrates the relationship between the wavelength and the highest degree of the SrO NPs made from *S. monoica.* Within a particular wavelength of 630 nm, the *S. monoica-derived* SrO_ _NPs showed a peak absorbance of 1.4, inhibiting a maximum efficiency of 1.4 at a frequency of 600nm.

Anti-inflammatory activity

**Figure 4:**
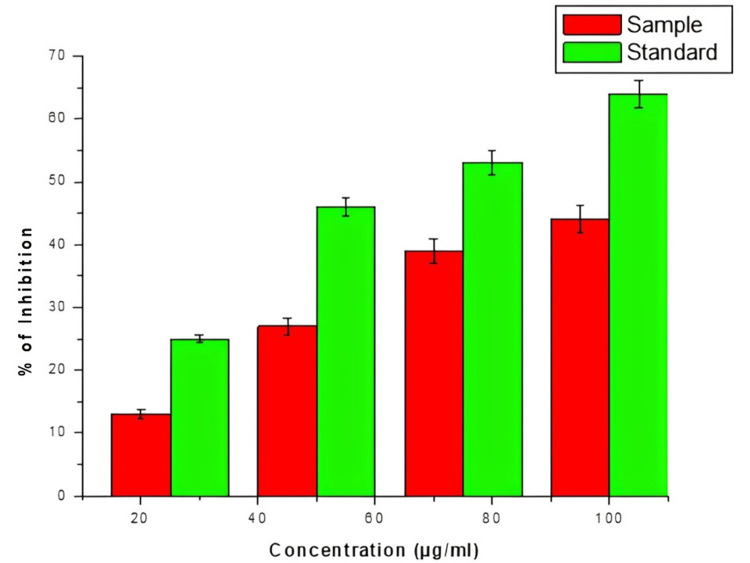
Anti-inflammatory activity of S. monoica

Figure 4 shows the percentage anti-inflammatory activity of *S. monoica* at various concentrations of 20 to 100 µg/mL. Among different concentrations of plant extract, the plant extract exhibits inhibition of concentration with gradually increasing concentration which increases the inhibition activity. Besides, compared to standard the extract activity was lower in ratio.

## Discussion

Recent research has demonstrated that plant extracts are capable of being utilized as a possible precursor to produce NPs. Alkaloids, flavonoids, saponins, steroids, tannins, and other naturally occurring chemicals are abundant in plants, as are other nutritious components. Numerous other metabolites found in the plant extract function as stabilizing and reducing agents throughout the bioreduction event that yield copper nanoparticles (Cu NPs) [22]. *C. tomentosum* contains powerful phytochemicals that are responsible for converting silver metal to silver nanoparticles (Ag NPs) by activating the surface plasmon resonance of synthetic Ag NPs. Using UV-visible spectroscopy, the bioreduction of Ag^+^ ions was detected in the solution of AgNO_3_ into silver nanoparticles from *C. tomentosum* phytochemicals [22, 23]. Au, Ag, and Fe NPs are just some of the metal nanoparticles that have been applied widely in medicine. AgNPs are employed for medication administration, while gold nanoparticles (AuNPs) are used for bioimaging and photothermal treatment, microbiological infection growth restriction, cancer treatment, and wound dressing. Zinc nanoparticles (ZnNPs) have lately been exploited as antibacterial and anticancer agents, and copper-based NPs have also been utilized in a variety of biomedical applications [22-24].

NPs have a profound effect on civilization [25]. The effectiveness of NPs in biomedical applications was tested [26]. In order to reduce the usage of such dangerous chemicals, the green synthesis technique was developed and is being used. The primary factor that has been shown in these studies with encouraging antibacterial results is the use of strontium and tea, which were used in this investigation. When certain bacteria come into contact with a combination of strontium and tea, the ions produced by the copper surface induce internal oxidative stress in the bacterial cell wall, which results in the death of the bacteria. Even though specialists have long been aware of this occurrence, their interest in it has recently grown [27].

*M. oleifera* leaf extract was successfully used as a lowering substance for the green production of lanthanum nanoparticles (La_2_O_3_ NPs) [28]. It is well known to have strong anti-inflammatory properties [29]. The focus of the study has recently shifted to strontium oxide nanoparticles (SrO NPs) because of their mysterious characteristics and potential uses. The employment of toxic chemicals, complicated equipment, and harsh experimental conditions are just a few of the numerous drawbacks of physicochemical strategies. An eco-friendly and sustainable method for producing SrO NPs from biogenic sources is a modern trend that may effectively substitute traditional methods. This paper examines all aspects that help the reader comprehend all-natural methods of using SrO NPs for a wide range of applications with lower toxicity concerns [30]. By delaying the growth of tumor cells, the Ag NPs made from *S. monoica* function as anticancer agents. In previous studies, there are reports of SrO NPs having cytotoxic effects on Hep2 cells, effectively preventing the growth of tumors while posing little danger to healthy cells. This could be because of their known inhibitory actions in several signaling cascades that are involved in the etiology and development of disease, which are as yet not understood [8,30].

Limitations

Overcoming these limitations through rigorous experimental design, thorough characterization, and careful evaluation of potential applications and safety concerns will be essential to the advancement of this field of study. Furthermore, working cooperatively with experts in the domains of biotechnology and nanotechnology can assist in overcoming some challenges. Further research will utilize in vivo animal models to examine the anti-inflammatory activity of the produced NPs. Enhancing the secure and efficient development of strontium NPs for a range of uses is feasible in addition to addressing these limitations and conducting further research in these sectors about side effects or difficulties.

## Conclusions

There are different potentials from the extraction of plant species to synthesize NPs, but understanding the synthesis process is necessary to realize the full potential of this technology. The production of SrO NPs using the extract of *S. monoica* was investigated using vis-spectra at 630 nm and was successfully used to reduce the toxicity produced by chemical methods. The *S. monoica* extracts performed a lowering and sealing of the substance to generate the needed SrO NPs. In vitro, anti-inflammatory activity of SrO NPs strongly impacts the activity of inflammatory enzymes against protein denaturation inhibition tests. Several factors, such as temperature, pH, nature of the capping agent, and concentration of active compounds, play important roles in determining NP size and shape. In this study, it is concluded that there are anti-inflammatory properties of SrO NPs synthesized from* S. monoica* saltmarsh. In comparison with the standard drug, bovine serum albumin, results for this aqueous extract were positive.
